# Layered Silicate-Alginate Composite Particles for the pH-Mediated Release of Theophylline

**DOI:** 10.3390/ph13080182

**Published:** 2020-08-06

**Authors:** Uttom Nandi, Vivek Trivedi, Dennis Douroumis, Andrew P. Mendham, Nichola J. Coleman

**Affiliations:** 1 Faculty of Engineering and Science, University of Greenwich, Central Avenue, Chatham Maritime, Kent ME4 4TB, UK; d.douroumis@greenwich.ac.uk (D.D.); a.p.mendham@gre.ac.uk (A.P.M.); N.Coleman@greenwich.ac.uk (N.J.C.); 2 Medway School of Pharmacy, University of Kent, Central Avenue, Chatham Maritime, Kent ME4 4TB, UK

**Keywords:** Laponite, alginate, composite particles, theophylline, pH-dependent release

## Abstract

Numerous natural and synthetic clay minerals have proven to be excellent drug carriers for high drug-loaded and sustained release formulations due to their considerable ion exchange, adsorption, and swelling capacities. Moreover, the synthetic smectite clays have added advantages in terms of compositional purity and controlled cation exchange capacity in comparison to natural clays. This study involves the intercalation of theophylline (TP) in a synthetic clay, Laponite^®^ (LP), followed by the inclusion of the resulting intercalates into sodium alginate (SA) beads to achieve pH-controlled drug release. Maximum intercalated drug incorporation of 68 mg/g was obtained by ion exchange at pH 1.2 and confirmed by an increase in basal spacing of the clay from 12.9 to 15.5 Å. TP release from the binary LP-TP intercalates in simulated gastric fluid (SGF) and simulated intestinal fluid (SIF) was found to be 40% and 70%, respectively. LP-TP particles were also incorporated in an SA matrix via polymer crosslinking using CaCl_2(aq)_ to improve the pH selective release. The ternary polymer-clay-drug composite particles effectively prevented the release of TP at low pH in SGF and resulted in sustained release in SIF, with 40% dissolution within 120 min.

## 1. Introduction

Theophylline (TP), C_7_H_8_N_4_O_2_, is a methylxanthine derivative that has been in clinical use for more than 70 years [1]. TP is primarily used as a bronchodilator at high concentrations, but low dosages are also known to provide an anti-inflammatory effect. It is a weak nonselective inhibitor of phosphodiesterase enzymes (PDEs), which prevents the breakdown of cyclic adenosine monophosphate and cyclic guanosine monophosphate, resulting in the relaxation of airway smooth muscles. It is also a potent adenosine receptor antagonist and treats bronchoconstriction caused by histamine release due to the actions of adenosine on the airway mast cells [2,3]. These mechanisms, along with the interleukin-10 release promoted by TP, ameliorate the symptoms of asthma and chronic obstructive pulmonary disease (COPD) [4]. However, the therapeutic window of TP is narrow, usually 10–20 mg/L, below which the bronchodilation effect is minimal, and above 25 mg/L, any additional benefits outweigh potential side effects [5]. In this respect, adverse effects associated with frequent doses, such as headaches, nausea, vomiting, abdominal discomfort and restlessness, are very common. Accordingly, the maintenance of serum TP concentration within the therapeutic boundaries is absolutely crucial to the wellbeing of the patient.

Layered silicate clays are popular vehicles for controlled release systems for the oral administration of neutral and cationic drugs that require gastric protection. Intercalated drug-clay systems that are capable of achieving high drug loading and modulated release can improve the therapeutic efficacy of the drug, reduce side effects and enhance patient compliance [6]. During the past few decades, LP has found myriad applications in various industrial sectors such as mining, petroleum refining, home and personal care, pharmaceutics, agrochemicals, paints, polymers and coatings [7,8]. A particular advantage of LP over that of natural smectites is its predictable compositional purity and controlled ion-exchange capacity. 

**Figure 1:**
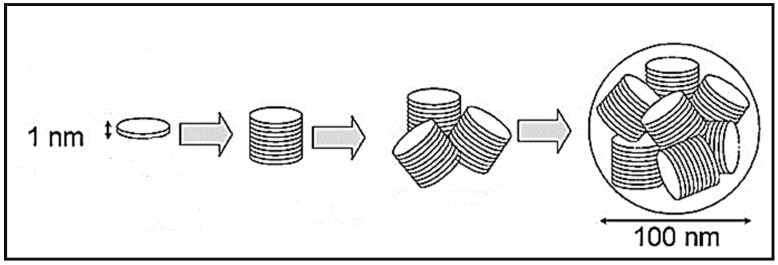
Schematic representation of an elementary disc crystal, stacked discs, and an aggregate and a cluster of stacked discs of Laponite^®^ [9].

Laponite^®^ (LP), Na_0.7_[(Si_8_Mg_5.5_Li_0.3_)O_20_(OH)_4_], is a synthetic analogue of a naturally-occurring smectite clay, hectorite [9,10,11]. Its crystal structure comprises a 2:1 layered system whose principal repeating unit is an octahedral magnesium oxide sheet bound between two tetrahedral silicate sheets, which are stacked to form nanodiscs measuring approximately 25 nm in diameter and 1 nm in height (Figure 1) [12]. The partial substitution of Mg^2+^ for Li^+^ within the octahedral magnesia layers confers a negative charge on the lattice that is balanced by ion-exchangeable interlayer Na^+^ ions [13]. Water molecules are also present within the interlayer spacing. Under selected conditions, the individual 2:1 silicate-magnesia-silicate layers can be entirely exfoliated (i.e., delaminated) into individual discs or expanded to accommodate large neutral or cationic species [14].

The principal objective of this study is to determine whether the intercalation of TP in LP will provide gastric protection and preferential release of the drug under intestinal conditions. The uptake kinetics of the cationic form of the drug by the clay in aqueous suspension at pH 1.2 was monitored by UV-vis spectroscopy as functions of LP and TP concentrations. The subsequent drug-clay interactions of the recovered solids were studied by powder X-ray diffraction analysis (XRD), Fourier transform infrared spectroscopy (FTIR), differential scanning calorimetry (DSC) and scanning electron microscopy (SEM). The LP-TP intercalates were further incorporated in a sodium alginate (SA) matrix to improve the pH specific dissolution of the drug. The release profiles of TP from the binary drug-clay hybrid and the ternary LP-TP-SA formulation were obtained in simulated gastric fluid (SGF) at pH 1.2 and simulated intestinal fluid (SIF) at pH 6.8. 

## 2. Results and Discussion

### 2.1. Uptake of Theophylline by Laponite^®^

**Figure 2:**
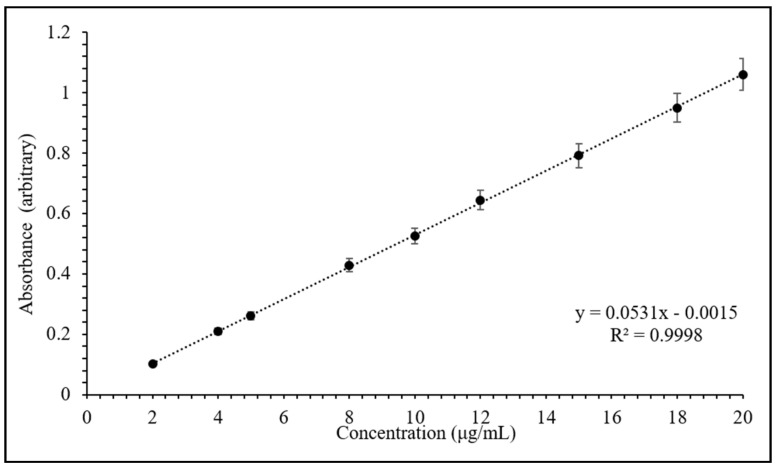
Calibration curve of theophylline (TP) in 0.1 N HCl at 270 nm. Values expressed are means with standard deviations as error bars, *n* = 3.

In this study, the drug content was monitored at 270 nm by UV-vis spectroscopic analysis using a calibration curve, as presented in Figure 2. The calibration curve is linear in the concentrations between 2 and 20 µg/mL, with a correlation coefficient (*R^2^*) of 0.9998 (as shown in Figure 2).

**Figure 3:**
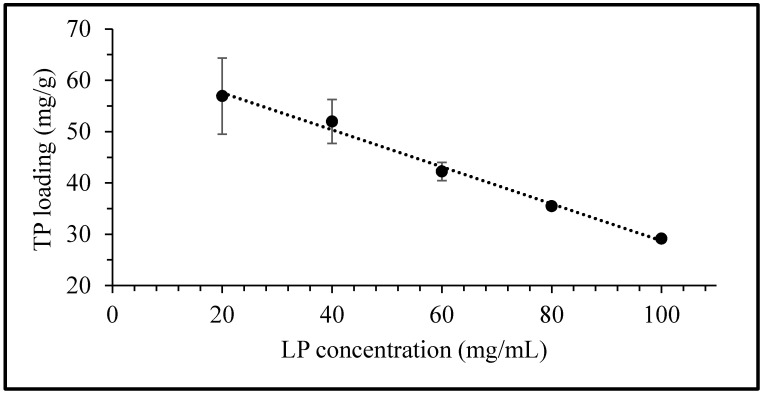
Equilibrium uptake of TP by Laponite® (LP) as a function of LP concentration. Values expressed are means with standard deviations as error bars, *n* = 3.

The equilibrium uptake of TP as a function of LP concentration is presented in Figure 3. The quantity of TP taken up per unit mass of LP is observed to decrease linearly with an increase in clay concentration. The equilibrium uptake of the drug at a fixed TP concentration of 3 mg/mL in 20, 40, 60, 80 and 100 mg/mL of LP was determined to be 57, 52, 42, 35 and 29 mg/g, respectively. The rectilinear character of the curve suggests that the maximum equilibrium concentration for interaction between both clay and drug has yet to be reached. This indicates that the ultimate sorption potential of the LP clay exceeds the maximum value obtained here (i.e., 57 mg/g) and that it could potentially accommodate even higher quantities of TP. Hence, further equilibrium uptake studies at LP concentrations of 20 and 40 mg/mL were also performed with varying drug concentrations up to 6 mg/mL. 

**Figure 4:**
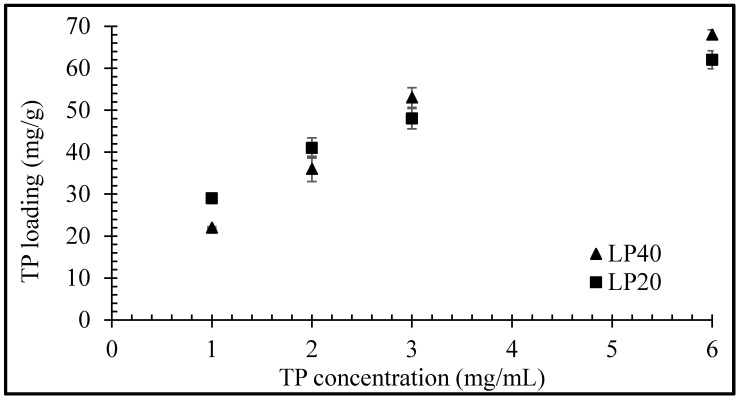
Equilibrium uptake of TP by LP as a function of TP concentration. Values expressed are means with standard deviations as error bars, *n* = 3.

Equilibrium uptake of TP by LP (for LP concentrations of 20 and 40 mg/mL) as a function of TP concentration is plotted in Figure 4. The quantity of adsorbed drug per unit mass of clay increased nonlinearly with increasing TP concentration, with maximum uptakes of 62 and 68 mg/g observed, respectively, for LP concentrations of 20 and 40 mg/mL with a drug concentration of 6 mg/mL. The main mechanism for drug uptake into clay is through intercalation, but a low percentage of TP molecules may also be able to adsorb on the clay surface that is unable to intercalate in the clay particles’ layers [15]. The higher probability of surface adsorption of drug molecules at 20 mg/mL can lead to slightly larger total drug entrapment values (Figure 4) at certain concentrations before the equilibrium is achieved.

**Figure 5:**
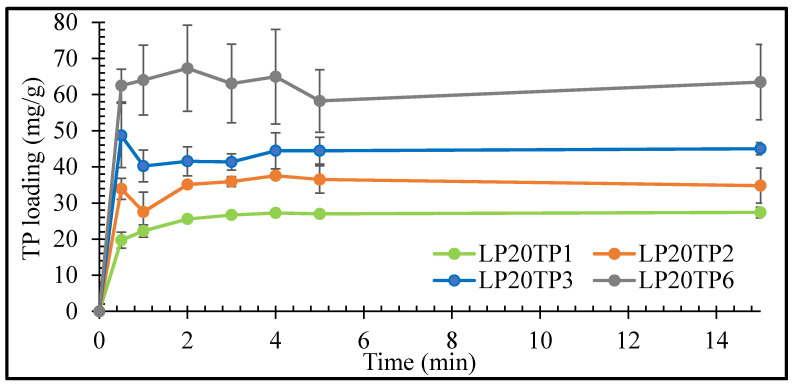
Uptake kinetics of TP by LP with 20 mg/mL LP. Values expressed are means with standard deviations as error bars, *n* = 3.

**Figure 6:**
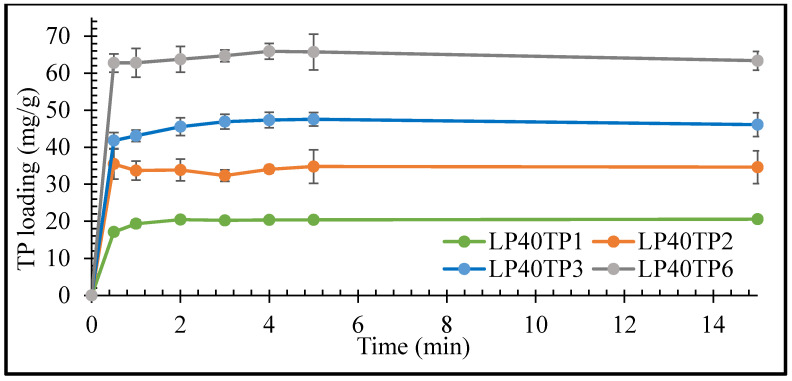
Uptake kinetics of TP by LP with 40 mg/mL LP. Values expressed are means with standard deviations as error bars, *n* = 3.

The rates of uptake of TP by the LP clay at pH 1.2 as functions of TP concentration are plotted in Figure 5 and Figure 6 for LP concentrations of 20 and 40 mg/mL, respectively. 

The initial rate of uptake of TP by LP was seen to increase with increasing drug concentration, with maximum uptake being achieved within 1 min irrespective of the drug or clay concentrations (Figure 5 and Figure 6). The prompt uptake of TP by LP is indicative of electrostatic interaction between the protonated cationic form of the drug and the negatively charged discs of clay at pH 1.2 [16,17]. This type of rapid exchange of the interlayer cations for the protonated form of TP has been previously demonstrated in a study of the adsorption of TP on natural smectite clay [18]. In addition to electrostatic interactions, other binding forces such as Van der Waals, hydrogen bonding and possible chemisorption may also take place between the drug and the nanoclay [17,19].

Results of the intercalation study demonstrated that TP interaction was variable and nonreproducible at 6 mg/mL of drug concentration. The percentage of intercalated TP into LP steadily decreased as the concentration of TP used in the bulk increased. The percentage uptake of TP after 15 min was 85%, 74%, 57% and 41% for 1, 2, 3 and 6 mg/mL TP, respectively, in an intercalation media containing 40 mg/mL of LP. The increase in TP concentration reflects an increase in the number of TP molecules that are competing for intercalation between the clay’s layers, i.e., the higher the number of available TP molecules to intercalate, the lower the percentage of them that will be intercalated. The large number of competing TP molecules can also lead to simultaneous intercalation and deintercalation of the drug, resulting in variable and nonreproducible results [15]. Based on the results here, LP-TP intercalates for further analysis and drug release were prepared with clay and drug concentrations of 40 mg/mL and 3 mg/mL, respectively, as these concentrations resulted in the most uniform drug-clay interactions.

### 2.2. Characterisation

**Figure 7:**
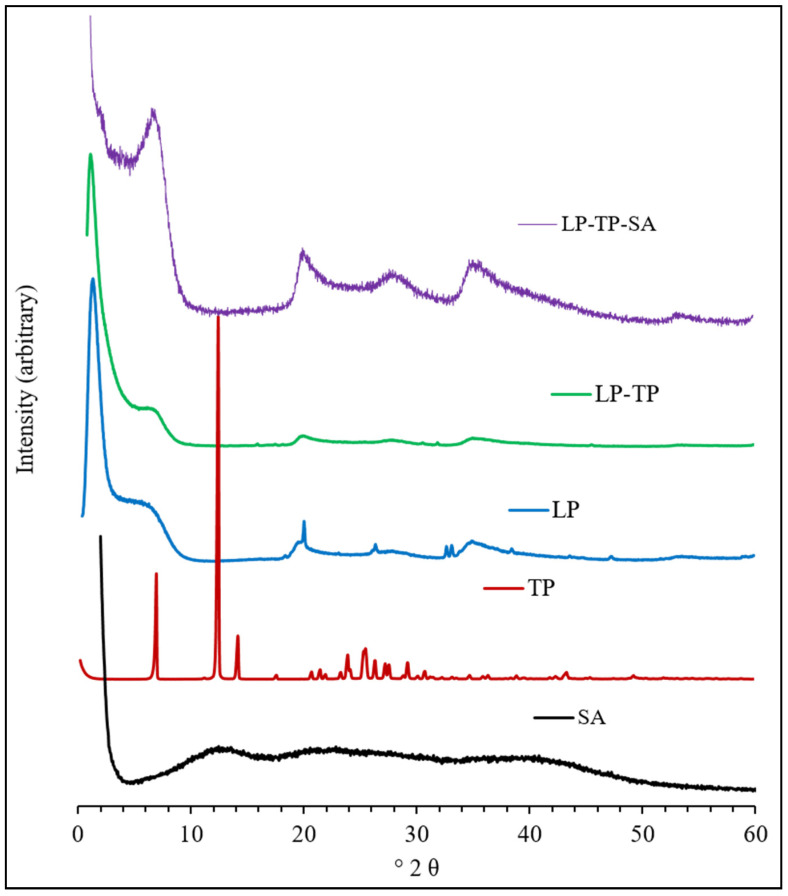
Diffractograms of sodium alginate (SA), TP, LP, LP-TP and LP-TP-SA.

The PXRD diffractograms of LP, TP, SA, the binary drug-clay hybrid (LP-TP) and the ternary drug-clay-alginate formulation (LP-TP-SA) are presented in Figure 7. 

The diffractogram of pure LP is characterised by reflections at 6.8°, 20.1°, 26.4°, 35.4°, 61.3° and 71.4°. The broad basal reflection at 6.8° denotes poor organisation of the clay layers in the *c*-axis direction, with a mean interlayer spacing of 12.9 Å. The diffraction pattern of TP closely resembles those reported in the literature and indicates that the material used in this study is pure crystalline anhydrous TP [18]. 

Conversely, reflections of TP were not present in the diffractogram of the binary LP-TP hybrid, which demonstrated that it was present in an amorphous form. An increase in the basal spacing of LP from 12.9 to 14.4 Å, following uptake of TP, confirmed that the drug was intercalated between the clay sheets [20,21]. No other changes in the diffraction pattern of LP were observed, which indicated that the crystalline structure of the individual LP sheets remained unaffected after drug intercalation. 

The diffraction pattern of sodium alginate used in this study had no distinct reflections, indicating that this polymer was amorphous. The ternary LP-TP-SA composite showed peaks similar to those of the pure LP, demonstrating that in-situ intercalation of alginate into the clay did not occur during the formation of the beads. 

**Figure 8:**
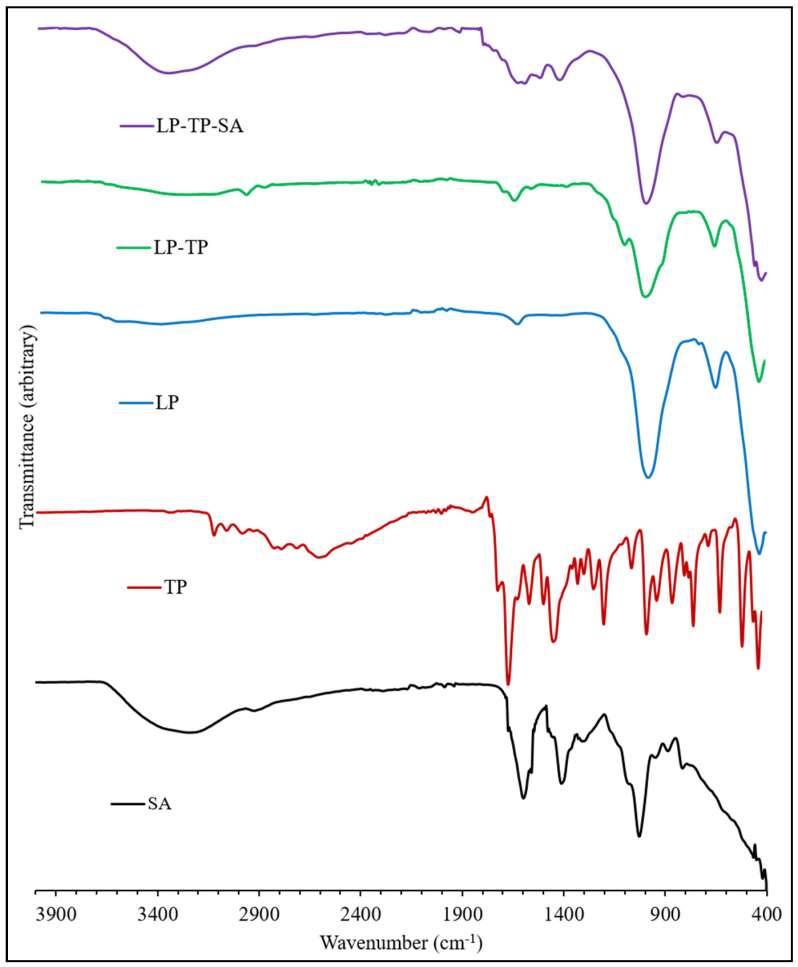
ATR-FTIR spectra of TP, LP, LP-TP and LP-TP-SA.

ATR-FTIR spectra of LP, TP, SA, LP-TP and LP-TP-SA are shown in Figure 8. The spectrum of LP shows a broad peak centred at 3445 cm^−1^ that arises from the O-H stretching vibrations of structural silanol groups (-Si-OH) and interlayer water molecules; the band at 1640 cm^−1^ is assigned to the bending modes of water [22,23]. The sorption band at 990 cm^−1^ is due to the Si-O stretching vibrations, and the bands at 650 and 434 cm^−1^ correspond to Mg-O-Mg and Si-O-Mg bending.

The FTIR spectrum of TP closely resembles those in the literature [17]. Bands in the range 3440 to 2460 cm^−1^ arise from the stretching modes of the N-H group and also the C-H bonds. Characteristic carbonyl (C=O) stretching occurs at 1710 and 1665 cm^−1^; the signal at 1565 cm^−1^ is assigned to amine N-H stretching. The aliphatic and aromatic conjugation of TP at 2988–3120 cm^−1^ was present in the spectrum of the binary LP-TP hybrid but was found to be shifted to 2976 cm^−1^ for the cationic intercalated form of the drug. 

The FTIR spectrum of sodium alginate, a linear copolymer of mannuronic and guluronic acids, presents a very broad band centred around 3250 cm^−1^, arising from stretching vibrations of the hydrogen-bonded -OH groups, -CH_2_ stretching modes at ~2900 cm^−1^ and characteristic asymmetric and symmetric vibrations of the carboxylate salt at 1610 and 1415 cm^−1^, respectively [24]. C-O stretching vibrations from the carboxylate groups and pyranose rings are also present at 1030 cm^−1^, with a shoulder at 1080 cm^−1^. In the spectrum of the ternary LP-TP-SA formulation, the narrowing of the -OH stretching vibration was indicative of the chelation with the crosslinking Ca^2+^ ions. The anticipated signals from the drug, clay and polymer within the ternary system were observed in the FTIR spectrum, although diagnostic analysis regarding the interactions between the components was not possible owing to the overlapping nature of the bands. 

**Figure 9:**
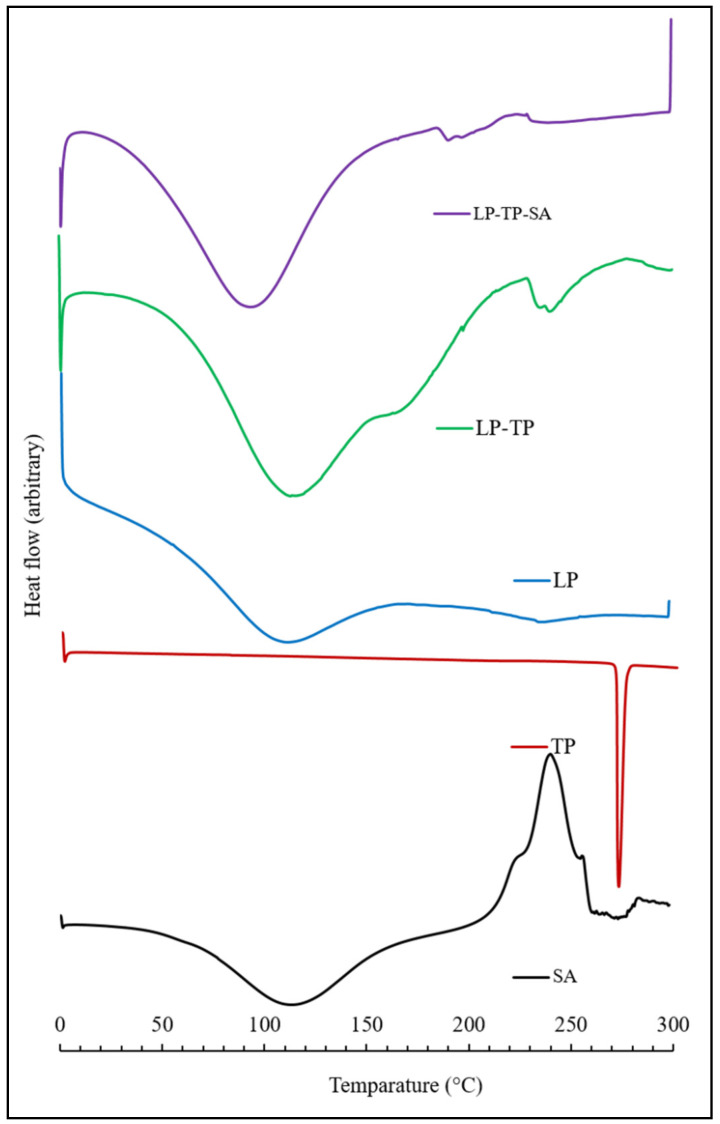
Thermograms of SA, TP, LP, LP-TP and LP-TP-SA.

The DSC thermogram of LP is characterised by two very broad endothermic peaks centred around 115 and 240 °C, corresponding to the loss of physisorbed and chemisorbed water, respectively (Figure 9) [25,26]. The melting temperature of TP was found to be 271 °C, which corresponds well with the values reported in the literature [18,27]. DSC analysis confirmed the absence of a TP melting peak within the binary LP-TP hybrid, demonstrating that the intercalated drug was present in an amorphous form (Figure 9) [18]. The endotherm corresponding to the loss of bound water from the LP-TP hybrid is also sharper and better defined than that of the original LP clay, indicating that the interlayer water is more uniformly adsorbed within the intercalated drug-clay system. 

As expected, the DSC thermogram of sodium alginate shows a broad endotherm at 115 °C, which is attributed to water loss, and also an exothermic event at 240 °C, denoting thermal decomposition [28]. The thermal profile of the LP-TP-SA system is indicative of free and bound water loss (at 100 and 200 °C, respectively) and also the decomposition of the polymer at 230 °C. The endotherms associated with the dehydration of free and bound water from the ternary crosslinked composite appear at lower temperatures than those of the pure sodium alginate and clay, although the reason for this is not presently known. 

**Figure 10:**
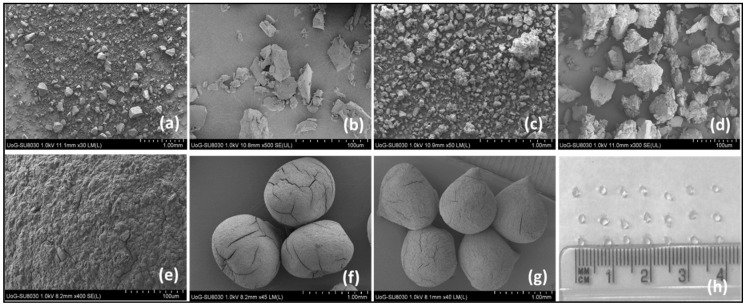
Secondary electron images of (**a**,**b**) LP, (**c**,**d**) LP-TP, (**e**) LP-TP-SA bead surface, (**f**,**g**) LP-TP-SA dried beads, and (**h**) digital photograph of LP-TP-SA beads.

A digital photographic image of freshly prepared composite beads, along with SEM micrographs of the pure clay, binary intercalates and dried beads, are presented in Figure 10. The freshly prepared LP-TP-SA beads were translucent, discrete, uniformly-sized spheres between 0.9 and 1.1 mm in diameter that shrank to between 0.60 and 0.65 mm after drying. Drying also resulted in a textured surface, with superficial cracks and cervices caused by water evaporation and a change in morphology from spheroidal to ellipsoidal and a mean aspect ratio of 1.15 ± 0.01 (as indicated in Figure 10) [29,30].

### 2.3. In Vitro Drug Release from LP-TP and LP-TP-SA

**Figure 11:**
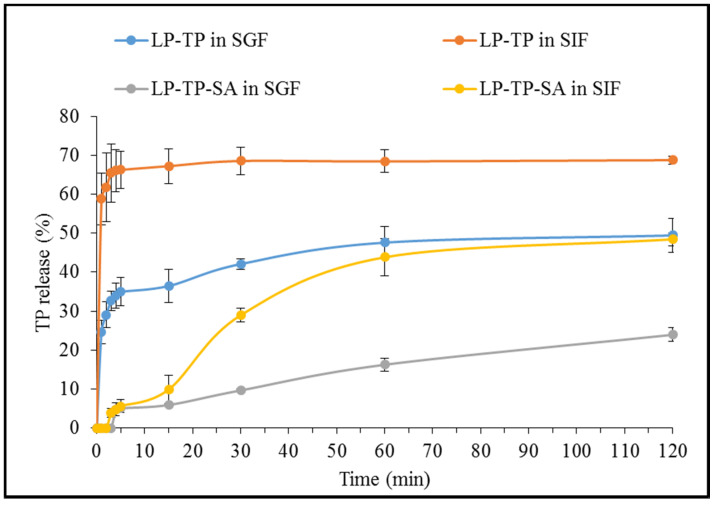
Dissolution profiles of TP from LP-TP and LP-TP-SA. Values expressed are means with standard deviations as error bars, *n* = 3.

The TP release from the binary LP-TP intercalates was found to be significantly faster and more extensive than that from the ternary LP-TP-SA composite beads in both SGF and SIF (as demonstrated in Figure 11). 

The LP-TP intercalates showed a burst release in SGF, reaching 35% within first 5 min, whereas the LP-TP-SA beads resulted in a modulated drug release of only 5% in the same timeframe. The extent of drug release was also considerably lower in SGF from the composite beads, i.e., 23% in comparison with 50% from drug-clay intercalates after 120 min. The erosion of LP and substitution of TP with H^+^ ions are considered to be critical factors that influence the drug release in an acidic media [20]. The presence of crosslinked SA around intercalates limits the diffusion of the dissolution media inside the composite particles, restricting the drug substitution and release in the acidic environment [31]. Alginates do not swell under acidic conditions and are rather known to shrink, resulting in limited or no drug release in the gastric environment. 

TP release from the binary intercalates was notably rapid in SIF, reaching a plateau of 68% within 15 min, whereas drug release from LP-TP-SA was considerably slower, reaching 50% after 120 min. Visually, the beads appeared to be broken within the first hour in SIF but remained intact, and a clear, homogeneous solution was not obtained after two hours. There are numerous reports suggesting that the high degree of the crosslinking and binding strength of calcium ions is the reason for the incomplete solubilisation of the calcium alginate network in SIF, which ultimately results in partial drug release [32,33,34]. LP is known to form a dense “house of cards” structure in alginate gels that is capable of encapsulating large quantities of drug within the matrix [7]. The combination of LP and alginate are able to provide pH-dependent sustained drug release by effectively controlling the rate of drug diffusion and water penetration that cannot be achieved from either intercalation in LP or incorporation in alginate gels alone [7]. Although drug release in this work was observed for 2 h, further sustained TP release can be expected to continue for a number of hours, based on the evidence present in the literature [15,35]. It may also be possible to achieve higher levels of drug release from these types of formulations by the incorporation of other large cationic molecules [36]. In addition, methacrylate polymers such as Eudragit^®^ contain carboxyl groups that remain ionised above pH 7, which can disturb the crosslinking and allow increased exposure to the dissolution media, resulting in the swelling of the polymer matrix and the release of the entrapped drug [37].

## 3. Materials and Methods

### 3.1. Materials

LP was supplied by BYK Additives (Wesel, Germany) and TP was kindly donated by BASF (Ludwigshafen am Rhein, Germany) in the form of a white anhydrous powder with a purity of >99%. SA was purchased from Sigma-Aldrich (Gillingham, UK). All other analytical grade reagents were obtained from Fisher Scientific (Loughborough, UK) and used as received.

### 3.2. Calibration Curve Preparation

The quantification of drug during intercalation, bead preparation and release studies was performed using UV-vis spectroscopy (Hitachi U-2900, Tokyo, Japan) at 270 nm. A standard stock solution of TP (200 μg/mL) was first prepared in 0.1 N HCl and then nine different concentration levels of calibration solutions (2 to 20 μg/mL) were freshly prepared by diluting suitable volumes of the stock standard solution in volumetric flasks. The absorbance of each solution was determined after filtration and plotted against concentration to determine the correlation coefficient and linear expression of the calibration curve.

### 3.3. Theophylline Intercalation and Drug Uptake Kinetics 

The equilibrium uptake of TP by LP was determined at 50 °C by varying the clay concentration from 20, 40, 60, 80 and 100 mg/mL against a fixed TP concentration of 3 mg/mL. In each case, an accurately weighed quantity of LP was dispersed for 30 min in 25 mL of 0.1 N HCl at pH 1.2 under sonication to promote the swelling of the clay. Then, 75 mg of TP was added to the suspension, and the sample was rotated on a Stuart SB3 tube rotator for 24 h at 40 rpm. Similar studies were also conducted to determine TP uptake using fixed LP concentrations of 20 and 40 mg/mL at 1, 2, 3 and 6 mg/mL TP concentrations. The suspensions obtained after 24 h were centrifuged at 7500 rpm for 2 min and, subsequently, the TP concentration in the supernatant liquor was determined via UV-vis spectroscopy at 270 nm upon filtration with 0.2 µm syringe filters. The experiments were performed in triplicate and the drug uptake in LP was calculated by subtracting the difference between the initial and final TP concentrations in the solutions. 

The rates of uptake of TP by LP were determined using 20 and 40 mg/mL clay suspensions and 1, 2, 3 and 6 mg/mL TP concentrations to obtain samples labelled as LP20TP1, LP20TP2, LP20TP3, LP20TP6, LP40TP1, LP40TP2, LP40TP3 and LP40TP6. Similar to the intercalation procedure described above, an accurately weighed quantity of TP was added to a suspension of swollen LP, and a small aliquot of dispersion media was removed to determine the drug content via UV-vis spectroscopy at 270 nm after filtration. The volume was kept constant to 25 mL throughout the experiment by the addition of fresh 0.1 N HCl after every step. 

### 3.4. Preparation of LP-TP-SA Composite Beads

LP-TP-SA composite beads containing the binary LP-TP intercalate were prepared using sample LP40TP3. For the preparation of LP-TP-SA, 1% *w/v* SA solution was first prepared in distilled water, with agitation at room temperature. A weighed quantity of LP-TP intercalate (1% *w/v*) was slowly added into the polymer solution, and the suspension was stirred for 30 min. The prepared suspension was then added into 0.5% *w/v* CaCl_2_ solution via syringe and 26 G needle from a fixed height of 3 cm from the surface of the CaCl_2_ solution. The LP-TP-SA beads were formed instantaneously upon contact with the CaCl_2_ solution and kept in the media for 12 h to allow further gelation and hardening. Thereafter, the beads were filtered, rinsed three times with 10 mL distilled water and dried in an oven at 50 °C for 24 h. The supernatant CaCl_2_ solution was collected after filtration and analysed via UV-vis spectroscopy at 270 nm to determine the loss of TP from the formulation during bead preparation, which was found to be 23 ± 2%.

### 3.5. Characterisation

The crystalline structures of the clay and drug were confirmed by powder X-ray diffraction analysis (XRD) using a Bruker D8 ADVANCE diffractometer (Bruker, Karlsruhe, Germany), with Cu Kα = 1.5406 Å at a step size of 0.02° in the 2θ range from 2° to 70° and a measuring time of 140 s per step. Fourier transform infrared (FTIR) spectra were acquired using a Perkin Elmer Spectrum Two spectrometer between 450 and 4000 cm^−1^ wavenumbers, with 10 scans at a resolution of 4 cm^−1^. Differential scanning calorimetry (DSC) was carried out using a Mettler-Toledo DSC823e calorimeter (Mettler-Toledo, Leicester, UK). Thermograms were collected on 2–3 mg of material in sealed, pierced aluminium pans between 25 and 300 °C at a heating rate of 10 °C min^−1^ under nitrogen. Scanning electron micrographs (SEMs) were obtained from uncoated samples attached to carbon tabs on a Hitachi SU8030 scanning electron microscope (Hitachi, Tokyo, Japan), with an accelerating voltage of 0.7 kV. 

### 3.6. In Vitro Release of Theophylline

The binary LP40TP3 intercalate and corresponding ternary LP-TP-SA composite beads were prepared according to the methods detailed in Section 3.3 and Section 3.4, respectively. In-vitro release of TP from both intercalates and beads was performed in SGF (pH 1.2) and SIF (pH 6.8). 

For the TP release procedures, SGF was prepared by dissolving 3.7 g of potassium chloride in 425 mL of 0.2 N HCl_(aq)_ that was made up to 1000 mL with deionised water [38]. For SIF, 6.85 g of potassium phosphate monobasic was dissolved in 1000 mL of water, and the pH was adjusted to 6.8 with small aliquots of 1 N NaOH_(aq)_ if required [39]. TP release profiles were obtained by suspending known quantities of the intercalates or beads containing 5 mg of drug in 200 mL of the selected dissolution medium. The dissolution medium was stirred at 100 rpm using a magnetic stirrer and maintained at 37.4 ± 0.2 °C in a water bath. Then, 3 mL of the supernatant was withdrawn at predetermined time points and analysed by UV-vis spectroscopy at 270 nm after passing through 0.45 μm polyethersulfone syringe filters. The drug release was calculated using the linear equation obtained from the calibration curve. The volume of the dissolution media was kept constant throughout the experiments by adding 3 mL of fresh dissolution media after each collection. Each experiment was carried out in triplicate. 

## 4. Conclusions

Theophylline was successfully intercalated into Laponite^®^ sheets to obtain a drug loading of 68 mg/g. Characterisation using powder X-ray diffraction analysis, Fourier transform infrared spectroscopy, differential scanning calorimetry and scanning electron microscopy confirmed that the drug had been intercalated into the clay in an amorphous form. The theophylline dissolution from the binary intercalated system in both simulated gastric and intestinal fluids showed a burst release which achieved a maximum value in less than 15 min. Alginate beads containing drug–clay intercalates were prepared to limit the drug release in the gastric environment and to prevent the burst release. The clay–drug–polymer beads limited the theophylline release to 23% in simulated gastric fluid and resulted in controlled and sustained drug release in simulated intestinal fluid. These findings suggest that Laponite^®^–alginate beads could be a potential platform for limiting theophylline absorption in the stomach, providing prolonged drug release in that small intestine that could potentially prevent drug fluctuations in serum concentration and reduce unwanted side effects.
